# The School Clinic
*The previous article appeared on March 16.


**Published:** 1912-03-30

**Authors:** 


### THE SCHOOL CLINIC.*

### II.—Treatment of Nose and Throat Cases at a School Clinic.

The majority of children referred with some enlargement of the tonsils and with adenoids do not need an operation. The treatment in these cases consists in correction of oral sepsis, and establishment of nasal respiration. The latter is to be obtained by breathing exercises steadily maintained over a considerable period of time. Further, many operations on adenoids fail in their effect unless these exercises be done. Therefore, some means of teaching the child nasal respiration is essential whether he be operated on or not. This continual attention cannot be given at a central clinic, but must be given at the individual schools. Something is already being done in this way, but more is necessary.

### School Treatment.

At first the children need more individual attention than is possible in a large class of children doing breathing exercises together. They speak the truth when they say they cannot breathe through the nose. They have never learnt to use their muscles in the proper way, and they must be taught it. This might be obtained by the appointment of a nurse to every school to see the treatment being carried out and such an appointment would fit in with the scheme suggested in the former paper, where the doctor was to see the patients a second time at the separate schools. The time of this nurse would not be taken up entirely by throat cases; she would also take charge of any continuous treatment of other children, such as skin cases; she would also see that the necessary children were got together for the visit of the doctor when he came. We do not think that there would be any difficulty in filling up the time of the nurse attached to each school. Any case that time proved to need an operation would then be referred back by the surgeon to the central clinic.

### The Operative Treatment.

In any district of our large cities there would still remain a large number of cases in which removal of the tonsils and adenoids would do good. In considering the operation, the fact that an appreciable number of more or less serious complications may arise must be borne in mind. A severe sore-throat lasting a week or so is the commonest, but not infrequently the exanthemata start within a short time of the operation, and though they might have had the illness anyhow, a sloughing ulcer on either side of the fauces is not a condition to be ignored in a case of diphtheria or scarlatina. Rheumatism in its subacute forms, including chorea, is also likely to be lit up by the operation. Lastly, acute infections passing further down the respiratory tract and giving rise to pneumonia, or out along the Eustachian tube to cause an otitis media, may also occur.

These complications are all the more likely to occur when the child goes out of doors within a couple of hours of the operation. This risk may be taken on a warm day in spring or summer, but it is not safe to operate on the children and send them home the same day during the winter months from October to March. In organising a clinic, therefore, the operative treatment should be reserved for the summer months, and even then a bed should be provided to keep a child in for the night if need be, or an ambulance should be obtainable for conveyance.

### Summary.

According to the scheme outlined in these articles, the parents would bring the children found defective to a clinic which would serve twenty or thirty schools and at which one or two throat surgeons would attend.

Notes would be taken, and a certain course of treatment prescribed. The notes would then be sent to the nurse of the school to which the child belonged, who would carry out the treatment ordered. The surgeon would then attend once a month or oftener at the school, and would re-examine the children to see how they were getting on. The examinations at the central clinic would be done as far as possible in the winter, so as to leave the rooms vacant for operative work in the summer.

By this time cases which undoubtedly needed operation would be sorted out from those which did not need them; and after the operation the breathing exercises would be recontinued by the nurse at the school. An exception may be taken to such a scheme on the ground of expense. But the nation has approved of the principle of treating these cases, and the efficient way must in the end be the cheapest. Such a central throat clinic with its interdependent school clinics and nurses, would be of no use without an associated dental clinic in the same area.

* The previous article appeared on March 16.

